# Multicentre Evaluation of Cepheid Xpert^®^ Research-Use-Only Panel for Molecular Rapid Detection of Gastrointestinal Pathogens

**DOI:** 10.3390/microorganisms14092011

**Published:** 2026-09-10

**Authors:** Olivier Dauwalder, Jan Kehrmann, Stéphane Corvec, Maroussia Roelens, Tiphaine Roussel-Gaillard, Edoardo Bixio, Jan Buer, Elisa Baillemont, Martin Prodel, Christophe Martinaud, Valeria Cento

**Affiliations:** 1 Plateau de Microbiologie 24/7, Institut des Agents Infectieux, Centre de Biologie et Pathologie Nord, Hospices Civils de Lyon & Université Claude Bernard Lyon 1, 69004 Lyon, France; 2 Institute of Medical Microbiology, University Hospital Essen, University of Duisburg-Essen, 45147 Essen, Germany; 3 Service de Bactériologie et des Contrôles Microbiologiques, INSERM, CNRS, Immunology and New Concepts in ImmunoTherapy, INCIT, UMR 1302/EMR6001 - Nantes Université & Univ. Angers & CHU Nantes, 44000 Nantes, France; 4 DALI, 42100 Saint-Etienne, Franceprodel@dali.science (M.P.); 5 Department of Biomedical Sciences, Humanitas University, Via Rita Levi Montalcini 4, 20090 Pieve, Italy; 6 Medical and Scientific Affairs, Cepheid, 81470 Maurens Scopont, France; 7 Microbiology and Clinical Virology, IRCCS Humanitas Research Hospital, 20089 Rozzano, Italy

**Keywords:** Xpert, gastrointestinal pathogens, diarrhoea, syndromic PCR panel, diagnostic stewardship

## Abstract

Rapid and accurate identification of enteric pathogens is essential for the management of acute infectious diarrhoeal diseases. Multiplex PCR panels have emerged as first-line screening tools to conventional stool culture by providing broad pathogen coverage and shorter turnaround times. This study evaluated the diagnostic performance of the Cepheid Xpert gastrointestinal (GI) research-use-only (RUO) panel in routine clinical practice across multiple European centres. A multicentre study was conducted in four hospitals across France, Germany and Italy. Stool specimens collected as part of routine care were tested using local standard-of-care (SoC) methods, including conventional stool culture, BDMax^®^ Enteric Panels, or BioFire FilmArray^®^ GI Panel, and subsequently analysed using the Cepheid Xpert GI RUO panel. A total of 11 bacterial, viral and parasitic enteropathogens were targeted. Discordant results underwent additional testing, and a consensus result based on a two-out-of-three agreement rule was used as the reference standard. Diagnostic performance metrics, agreement with SoC methods, microbiological findings and turnaround times were assessed. Of 508 samples, 115 (22.6%) were identified as positive for at least one pathogen according to the consensus result, including 12 samples with co-detection of more than one pathogen (10.4% of positive samples), resulting in a total of 128 pathogens detected. The most frequently detected pathogens were *Campylobacter* spp. (37 specimens), Norovirus (26 specimens), and *Salmonella* spp. (25 specimens). Compared with the consensus reference standard, Xpert achieved an overall sensitivity of 98% (95% CI: 94–100%), specificity of 100% (95% CI: 100–100%), positive predictive value of 95% (95% CI: 90–98%), and negative predictive value of 100% (95% CI: 100–100%). Sensitivity was ≥95% and specificity ≥99% for all evaluable targets. Agreement with routine diagnostic methods was high, with overall positive and negative percent agreements exceeding 90% and a Cohen’s kappa coefficient of 0.91. Median analytical turnaround time was 1 h 18 min for Xpert, compared with 3 h for BDMax, 1 h 15 min for BioFire and 48 h for conventional stool culture. The Cepheid Xpert GI (RUO) panel provided highly accurate detection of enteric pathogens across heterogeneous European diagnostic settings and reduced turnaround time by more than 95% compared with conventional stool culture. A future IVD version of this test could provide rapid and accurate detection of enteric pathogens for potential routine management of patients with suspected acute infectious gastroenteritis.

## 1. Introduction

Acute infectious diarrhoea remains a major global public health concern and is responsible for substantial morbidity and mortality worldwide, particularly among children under five years of age in low- and middle-income countries [1]. A wide range of bacterial, viral, and parasitic pathogens can cause gastrointestinal infections, making rapid and accurate etiological diagnosis essential for appropriate patient management, antimicrobial treatment onset, antibiotics conservation, prevention of complications, and implementation of public health measures [2].

Historically, conventional diagnostic approaches for enteric pathogens relied on stool culture, microscopy, and targeted biochemical or immunological tests. Although these methods have long represented the reference standard for pathogen detection, they are labour-intensive, time-consuming, of suboptimal sensitivity and often associated with prolonged turnaround times. In addition, conventional culture frequently requires several identifications, additional confirmatory testing, and enrichment broth, therefore potentially delaying diagnosis and therapeutic decisions, with results arriving mainly after the standard treatment duration of anti-infectious therapies, and having finally a very limited clinical value.

Over the past decade, advances in molecular diagnostics have transformed the microbiological investigation of diarrhoeal diseases. The implementation of syndromic multiplex PCR panels in clinical laboratories is increasing as they enable the simultaneous detection of multiple bacterial, viral and parasitic pathogens in stool specimens in a single assay. Several commercial gastrointestinal (GI) panels are currently available, including the BioFire FilmArray^®^ Gastrointestinal Panel (bioMérieux), the Verigene^®^ Enteric Pathogens Test (DiaSorin), the QIAstat-Dx^®^ Gastrointestinal Panel (Qiagen), the BDMax^®^ Enteric Panels (Waters), and the Allplex^TM^ Gastrointestinal Panel Assays (Seegene). Compared with conventional methods, these assays provide several advantages, including significantly reduced turnaround times, high sensitivity and specificity, improved negative predictive value, reduced laboratory workload, and the ability to identify coinfections [3,4,5,6,7]. These features are now part of official guidelines for gastrointestinal infections management in some countries [8,9,10], and may facilitate earlier implementation of targeted therapy and infection control interventions while limiting unnecessary antimicrobial use.

The Cepheid Xpert GI research-use-only (RUO) panel assay (Xpert) is a syndromic PCR panel that simultaneously detects 11 bacterial (n = 8), viral (n = 1) and parasitic (n = 2) enteric pathogens: *Salmonella* sp., *Shigella/Enteroinvasive Escherichia coli*, *Campylobacter (C. jejuni/C. coli)*, *Yersinia enterocolitica*, Norovirus, *Giardia* sp., *Cryptosporidium* sp., *Vibrio cholerae*, *Vibrio parahaemolyticus*, Shiga toxin 1 and Shiga toxin 2 of enterohemorrhagic *E. coli (EHEC)*. The purpose of this study was to evaluate the diagnostic accuracy of the Xpert panel for all aforementioned targets and compare the Xpert test performances to other approaches (including conventional stool culture but also other syndromic/multiplex PCR panels) using in routine care testing.

## 2. Materials and Methods

### 2.1. Sample Collection and Processing

This study was performed at four different hospitals in three European countries: the Hospices Civils de Lyon (France), the Centre Hospitalier Universitaire de Nantes (France), the Humanitas Research Hospital in Milan (Italy), and the Essen University Hospital (Germany).

Stool samples used in this study were consecutive residual samples (“leftover”), collected in Cary Blair medium as part of the routine care activities of the investigation centres, among inpatients, outpatients, emergency room patients, or patients admitted in other hospital wards in the first 72 h. No selection criteria based on age or clinical condition of patients were applied. As no additional procedures or interventions were performed for research purposes, this study did not constitute interventional human subjects research and therefore did not require review by an ethics committee. However, for some countries/hospitals, an information letter was sent to all included patients, allowing for them to express their opposition to participating in this study.

Collected samples were either transferred immediately to the laboratory or stored at 4 °C for 72 h at most (from sample collection to laboratory analysis start). Samples processed on the same day as receipt were analysed fresh at room temperature immediately following preparation. Samples that could not be tested on the day of receipt were brought back to room temperature prior to testing. Remaining biological material was subsequently transferred to long-term storage at −20 °C after analysis. Distinct Standard-of-Care (SoC) approaches for GI pathogen detection were implemented in each centre. Therefore, samples investigated at the Hospices Civils de Lyon and at the Essen University Hospital were screened for all 11 target pathogens (*Salmonella* sp., *Shigella* sp., *Campylobacter* sp., *Yersinia* sp., Norovirus, *Giardia*, *Cryptosporidium*, *V. cholerae*, *V. parahaemolyticus*, Shiga toxin 1 and Shiga toxin 2), using either BDMax^®^ extended Enteric Bacterial/Parasite/Viral Panel (Waters, Sparks, MD, hereafter BDMax; results for recorded for a selected subset of pathogens corresponding to the 11 targets detected by Xpert: *Salmonella* sp.; *Campylobacter* sp. including *C. jejuni* and *C. coli*; *Shigella* spp./EIEC; Shiga toxin 1 and 2; *Vibrio* sp., including *V. vulnificus*, *V. parahaemolyticus*, and *V. cholerae*; *Y. enterocolitica*; *Cryptosporidium parvum*; *Giardia lamblia*; and Norovirus) at the Hospices Civil de Lyon, or BioFire FilmArray^®^ GI Panel (bioMérieux SA, Marcy l’Étoile, France, hereafter BioFire; results for recorded for a selected subset of pathogens corresponding to the 11 targets detected by Xpert: *Salmonella* spp.; *Campylobacter* sp. including *C. jejuni*, *C. coli* and *C. upsaliensis*; *Shigella* spp./EIEC; Shiga toxin 1 and 2; *Vibrio* spp., including *V. vulnificus*, *V. parahaemolyticus*, and *V. cholerae* reported specifically; *Y. enterocolitica*; *Cryptosporidium*; *Giardia*; and Norovirus) at the Essen University Hospital. In contrast, stool culture was the SoC for enteric pathogen detection both at the Centre Hospitalier Universitaire de Nantes and at the Humanitas Research Hospital in Milan, with selected pathogens targeted (*Salmonella* spp., *Shigella* spp., *Campylobacter* spp. and *Yersinia* spp.).

Because SoC target coverage differed between centres, diagnostic performance analyses were performed on a per-sample, per-target basis, restricted to pathogen targets for which comparator testing was available within the local SoC and consensus-testing framework.

### 2.2. Resolution of Discordant Cases

Similar to other studies [3], in cases where SoC and Xpert results were discordant, a third test was performed, using a 2-out-of-3 rule to define the biological truth regarding enteric pathogen investigation, that result being referred to as the consensus in following analyses. At the Essen University Hospital, third discrepancy testing was performed using either culture on selective media, antigen testing, Xpert Norovirus or Seegene Allplex^TM^ GI-Virus Assay, depending on the target pathogen. At the Centre Hospitalier Universitaire de Nantes, consensus resolution was done using BDMax Enteric Bacterial Panel or BDMax Extended Enteric Bacterial Panel, while BioFire GI panel was used at the Hospices Civils de Lyon.

At the Humanitas Research Hospital, discordant samples for *Giardia* were resolved using the GI Parasitic PLUS ELITe MGB^®^ Kit (ELITechGroup S.p.A., Torino, Italy), while bacterial discordance were resolved via metagenomic sequencing using a MinION™ MK1D (Oxford Nanopore Technologies, Oxford, UK) and Rapid Barcoding Kit (SQK-RBK-114.96, Oxford Nanopore Technologies, Oxford, UK). The software MinKNOW (v25.09.16) was used for real-time basecalling (Dorado v7.11.2) (Q ≥ 10). Untargeted classification was performed running Kraken2 (v2.1.3) on the basecalled reads using the standard database. A target-directed classification was performed by mapping the basecalled reads to the specific reference genomes of the discordant targets: *Campylobacter coli* (GCA_009730395.1), *Campylobacter jejuni* (GCA_000009085.1), *Yersinia enterocolitca* (GCA_901472495.1), and *Stx1* (X07903.1). Alignment was conducted using minimap2 (v2.30) with parameters optimised for Oxford Nanopore reads (-ax map-ont). Alignments were sorted and indexed using SAMtools (v1.23.1), and mapping quality and coverage statistics were generated using QualiMap (v2.3). The following mapping thresholds were used: (i) a mean depth of coverage ≥ 3X, (ii) a mean mapping quality (MAPQ) ≥ 10, (iii) a coverage breadth ≥ 10% for the target genomes, and ≥80% for target genes.

### 2.3. Sample Size

No formal sample size calculation was performed. The number of specimens included reflects the availability of routine stool samples collected during the study period at the participating centres. This number was considered sufficient to provide stable estimates of the sensitivity and specificity for the most prevalent pathogens.

### 2.4. Data Analysis

First, descriptive analyses were performed to characterise the study population and microbiological results. Patient demographics, including age, sex, participating centres, and hospital clinical wards, were summarised using appropriate descriptive statistics, performed at the sample level (N = 508). Positive samples (as per the consensus result) were described according to their distribution across SoC test type and detected pathogens, and positivity rates were calculated for each pathogen. Cases of multiple pathogen detection within a single sample were further investigated. The analytical turnaround time from sample receipt at the workbench to result reporting was assessed for each diagnostic method, including Xpert, BioFire, BDMax, and conventional stool culture. Given the non-normal distribution of turnaround times, results are reported as median values with interquartile ranges (IQRs). No formal statistical comparison of analytical turnaround time between methods was performed, as turnaround times for syndromic/multiplex PCR platforms showed no variability within each platform.

Then, Xpert results from clinical specimens were compared to both SoC and consensus, on a per-target basis. Pathogen testing results for Shiga toxin 1 and Shiga toxin 2 were grouped together under the label “EHEC *Stx*1/2”, considering that the test was positive in case any of those two pathogens was detected. Similarly, testing results for *V. cholerae* and *V. parahaemolyticus* were grouped together under the “*Vibrio* spp.” label.

Diagnostic performance metrics, including sensitivity, specificity, positive predictive value (PPV) and negative predictive value (NPV), were assessed at the pathogen test level, by comparing each assay against the consensus result. Therefore, false negatives and false positives correspond to observations with discordance between the result of the considered method (culture, BDMax, BioFire or Xpert) and the consensus result, while true positives and true negatives correspond to observations with agreement between the result of the considered method and the consensus result. As a reminder, if we denote TP as true positives, FP as false positives, TN as true negatives and FN as false negatives, sensitivity corresponds to the proportion of true positive samples correctly identified by the assay (TP/(TP + FN)), specificity to the proportion of true negative samples correctly identified (TN/(TN + FP)), PPV to the probability that a positive result reflected the true presence of the pathogen (TP/(TP + FP)), and NPV to the probability that a negative result reflected its true absence (TN/(TN + FN)). Each sample contributed as one independent observation per investigated pathogen target, resulting in up to 2964 pathogen-specific analyses. Since not all target pathogens were investigated in each sample, the number of pathogen-specific tests is not strictly equal to the number of samples (N = 508) multiplied by 11 (the total number of investigated pathogens in that study). Furthermore, agreement between the Xpert assay and the SoC methods (overall and per SoC type) was assessed using positive percent agreement (PPA), negative percent agreement (NPA), and Cohen’s kappa coefficient [11]. For each of those pathogen-specific performance indicators, 95% confidence intervals (CIs) were calculated using the Wilson score method for binomial proportions, while for Kappa they were estimated using a non-parametric bootstrap approach with 500 resamples (kappa was calculated for each bootstrap dataset, and the 2.5th and 97.5th percentiles of the resulting distribution were used to define the confidence interval). In addition, overall diagnostic performance was also calculated, across all samples and all pathogens, with each sample–pathogen pair contributing equally. Associated 95% confidence intervals were obtained using a sample-level cluster bootstrap with 500 resamples, to account for within-sample correlation across pathogens. Results for PPA, NPA, and Cohen’s kappa are provided in the Appendix A. For the SoC testing methods, diagnosis performances were calculated both overall and per SoC type (molecular multiplex or conventional culture).

Discordant samples, defined as specimens for which either the SoC method or the Xpert assay yielded results inconsistent with the consensus, were reviewed separately.

Given the limited number of positive specimens at the individual centre level, no formal subgroup analysis by participating centre was performed.

All statistical analyses were performed using Python (version 3.14; Python Software Foundation, Beaverton, OR, USA) with the following libraries: scipy (version 1.17) for statistical testing, statsmodels (version 0.14) for confidence interval estimation, and pandas (version 3.0) for data management. Figures were generated using matplotlib (version 3.10) and seaborn (version 0.13).

## 3. Results

### 3.1. Description of the Study Population

**Figure A1:**
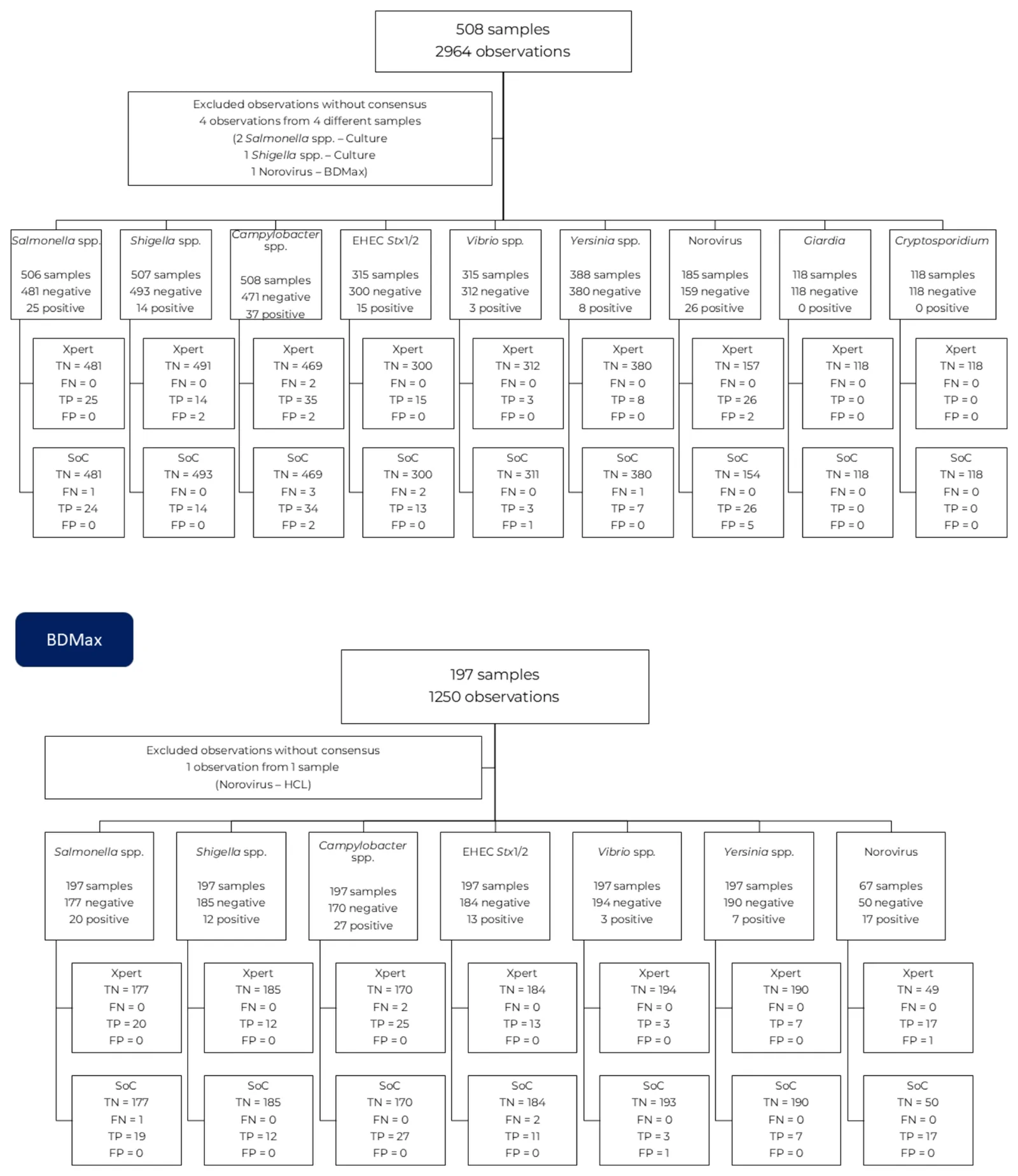

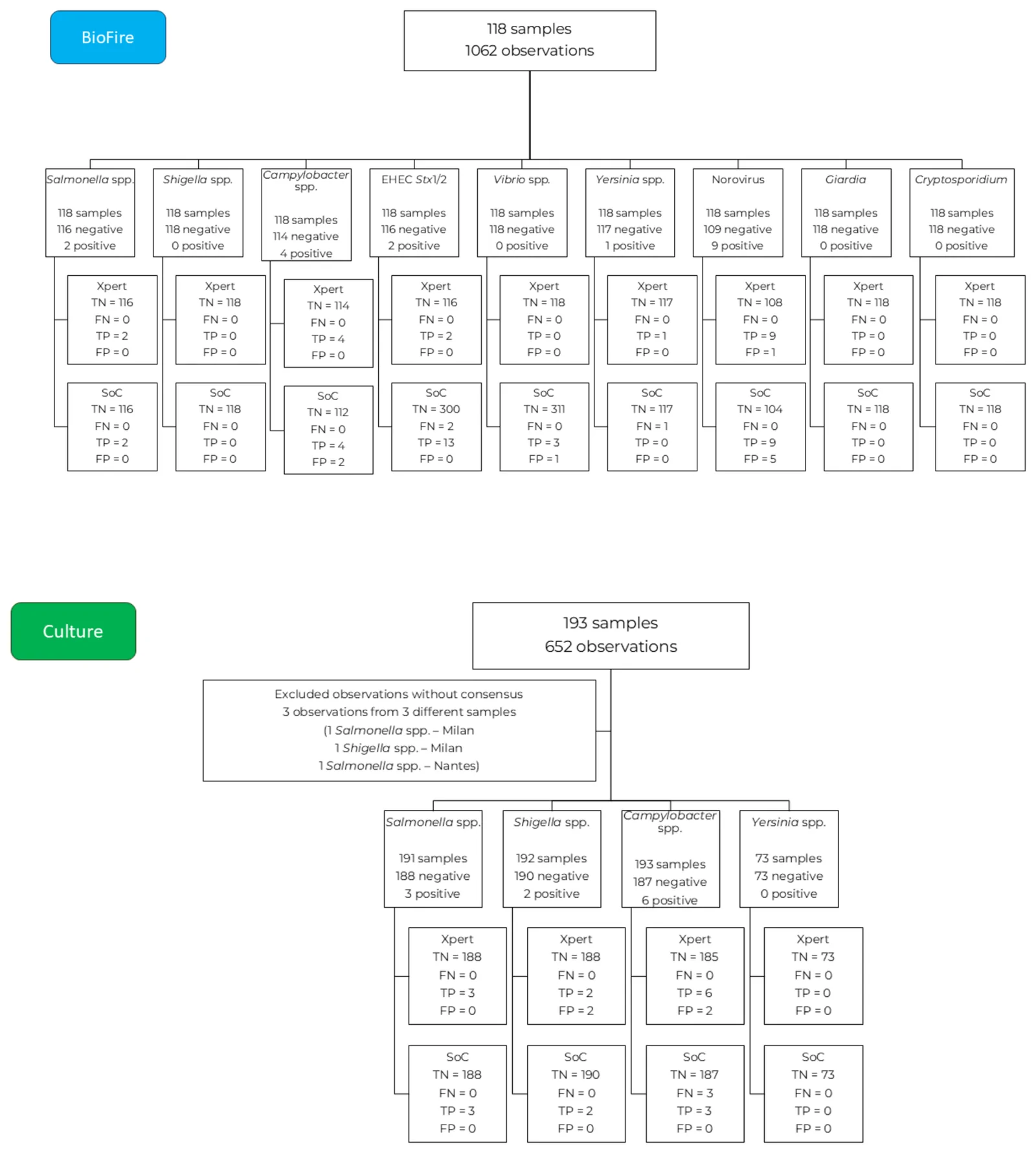
Flowchart of samples analysed, included unresolved observations, per pathogen and per test type (SoC or Xpert). Separate figures per SoC test type (BDMax, BioFire or culture) are also shown.

A total of 508 samples were included in the study, prospectively collected between 24 December 2025 and 31 March 2026, corresponding to a total of 2964 gastrointestinal diagnosis tests on a per-target basis (see Appendix A, Figure A1 and Table A1). Of note, for four of those 2964 tests, the biological consensus could not be retrieved, due to an insufficient amount of biological material to perform an additional GI detection test.

**Figure 1:**
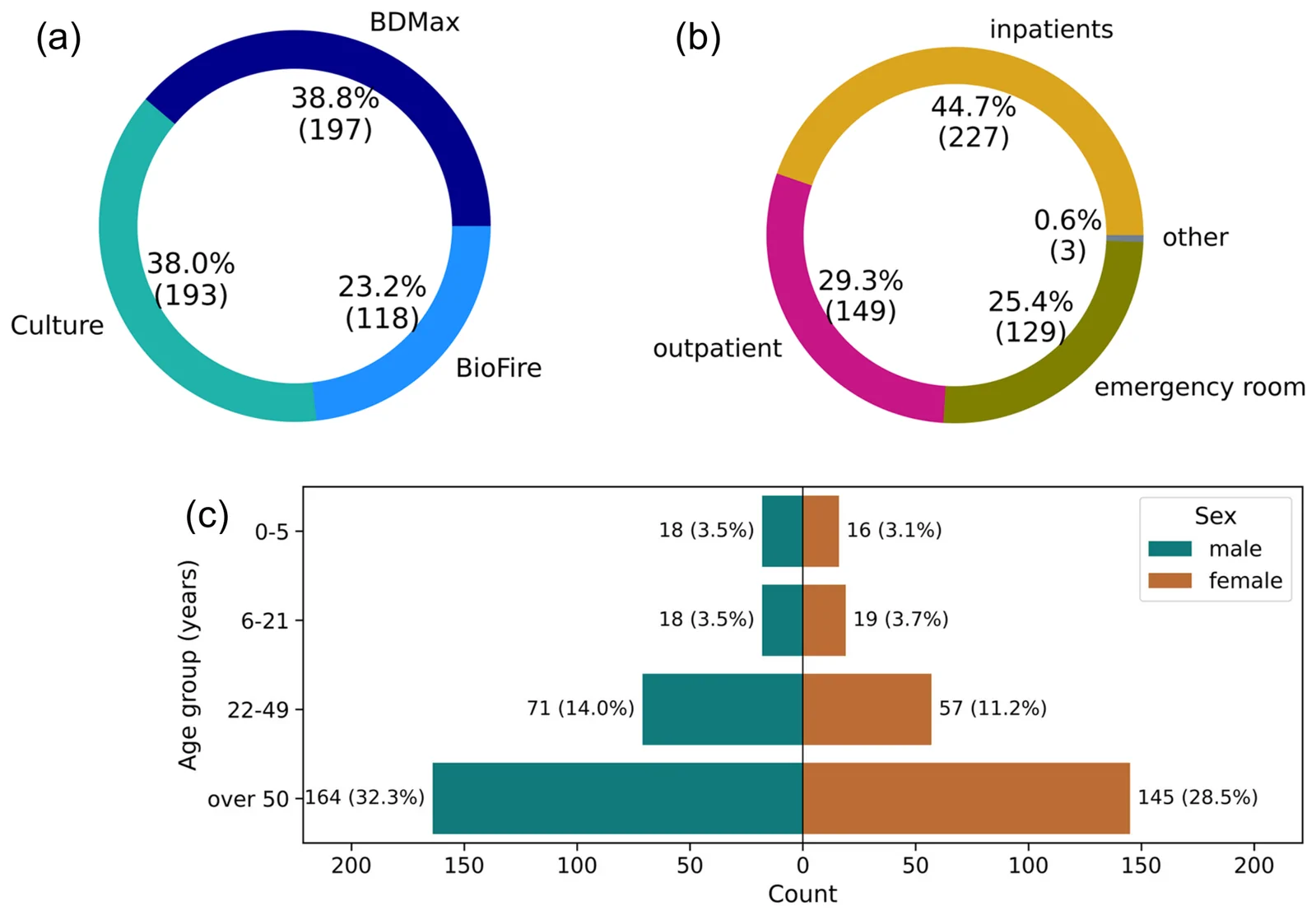
Description of the study population: (**a**) distribution of samples per SoC test type; (**b**) distribution of samples per collection ward; (**c**) distribution of samples per patient’s age and sex.

Among the 508 included samples, 197 (38.8%) were initially tested using BDMax as the SoC method, 118 (23.2%) with BioFire, and 193 (38.0%) by conventional stool culture (Figure 1a). Most specimens were obtained from inpatients (227/508, 44.7%), followed by outpatients (149/508, 29.3%) and patients presenting to the emergency department (129/508, 25.4%). Only three samples (0.6%) originated from other hospital units (Figure 1b). The study population was predominantly male, accounting for 271 patients (53.3%), and was largely composed of individuals aged 50 years or older (309 patients, 60.8%), as depicted in Figure 1c.

### 3.2. Description of Microbiological Results

**Figure 2:**
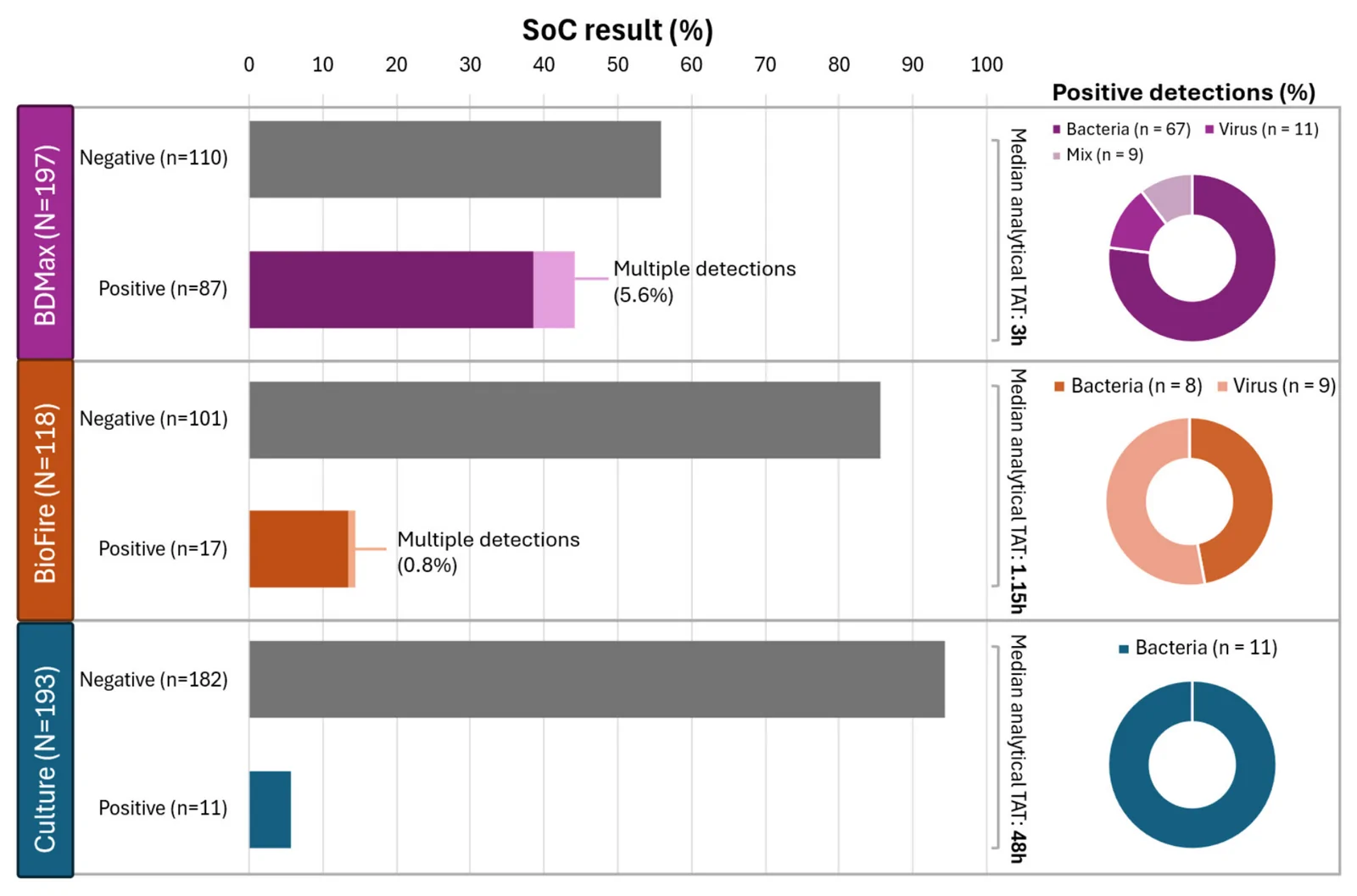
Overall consensus results for each SoC testing method.

**Figure 3:**
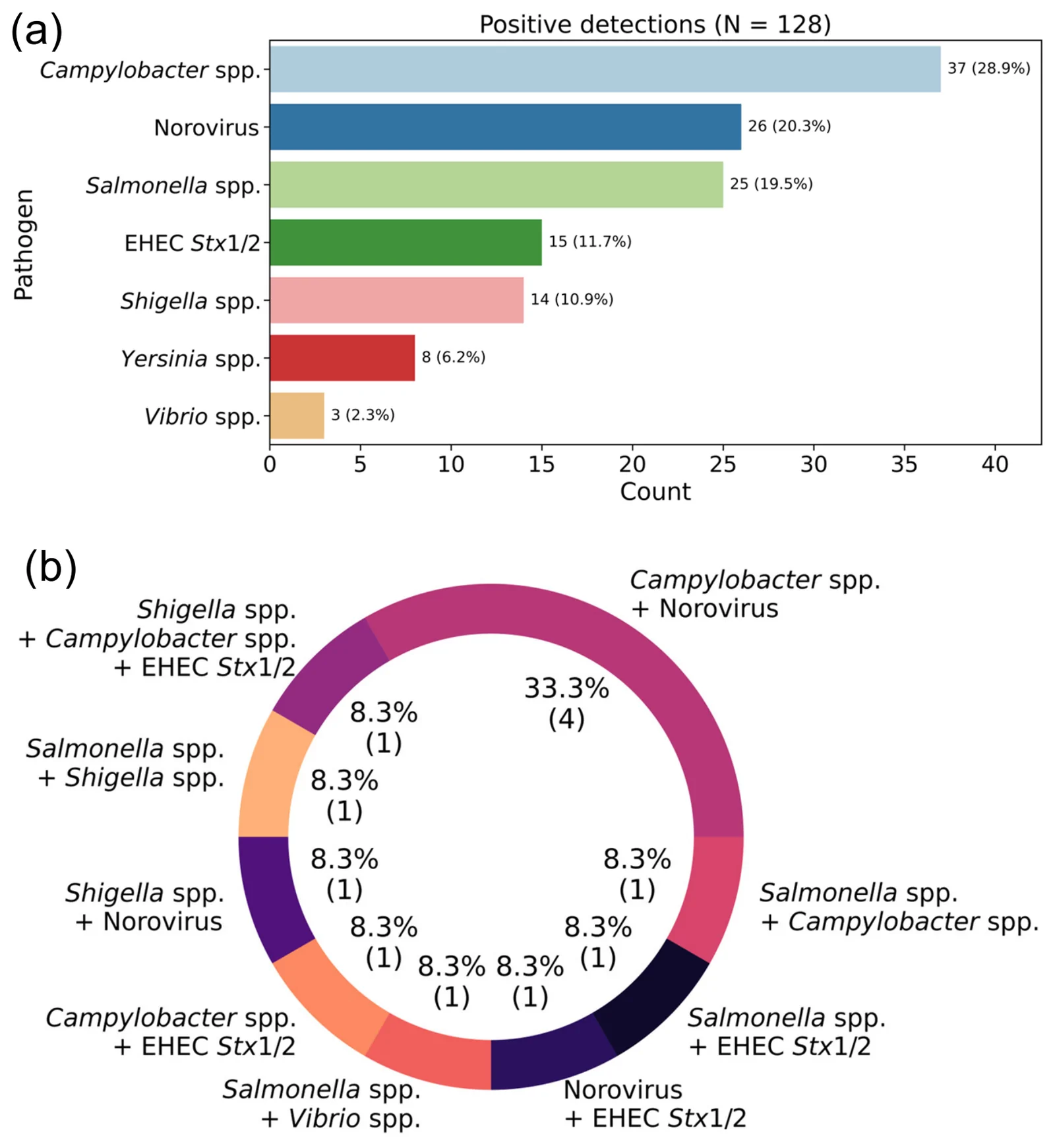
(**a**) Distribution of positive detections across investigated pathogens, as per the consensus result. (**b**) Distribution of pathogens among samples with co-detections (as per the consensus result).

Of 508 samples, 115 (22.6%) were identified as positive to at least one pathogen among the ones investigated in the current study (as per the consensus result). The overall consensus results for each SoC approach are shown in Figure 2. A total of 128 pathogens was detected during the study period. Figure 3a shows the distribution of positive detections across investigated pathogens. The most frequently detected pathogens were *Campylobacter* spp. (37 detections, 28.9% of positive detections), Norovirus (26 detections, 20.3%) and *Salmonella* spp. (25 detections, 19.5%). No sample positive to *Giardia* nor to *Cryptosporidium* was collected. A total of 12 samples had co-detection of more than one pathogen (10.4% of positive samples) (Figure 3b), with two pathogens detected in 11 samples (91.7% of co-detections) and only one with three pathogens detected (8.3% of co-detections). Most co-detections involve both *Campylobacter* spp. and Norovirus (four samples, 33.3% of samples with co-detection), those two pathogens being the most frequent ones in co-detection cases.

### 3.3. Analysis of Testing Turnaround Time

**Figure 4:**
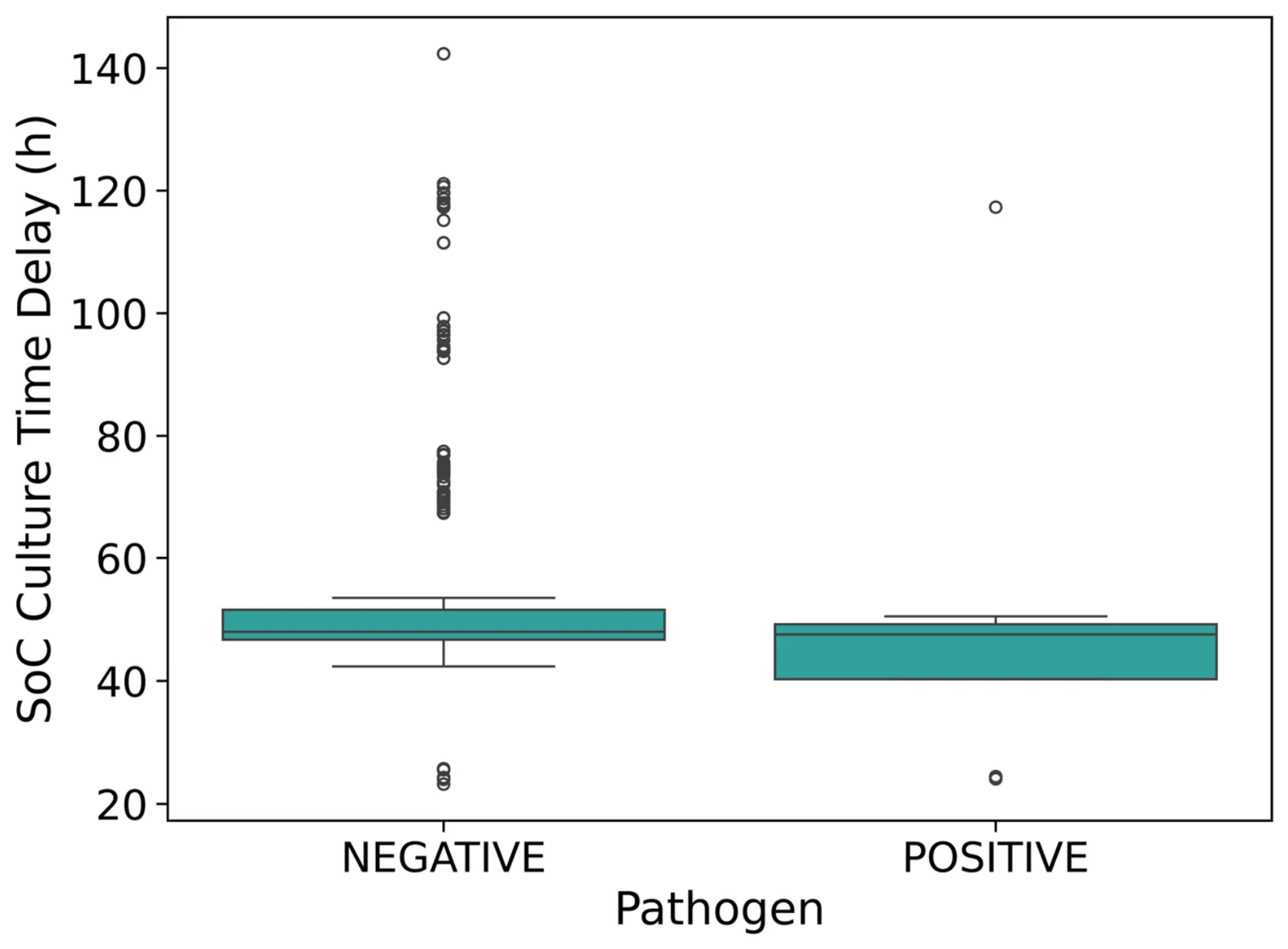
Analytical turnaround time to result reporting in conventional stool culture, stratified per test results. Of note, the outlier with turnaround time ~120 h among positive samples was confirmed, the prolonged time reflecting a real-world confirmatory workflow including additional identification steps.

The median analytical turnaround time from sample receipt to result reporting was 48 h for stool culture, 3 h for BDMax, 1 h 18 min for Xpert and 1 h 15 min for BioFire. Of note, this analytical time delay did not vary for a given syndromic/multiplex PCR panel (BDMax, BioFire, Xpert), as the diagnosis testing process is very standard in those cases. On the other hand, the time delay in conventional culture could vary significantly from one sample to another, particularly among negative specimens, with a few samples associated with delays of more than 80 h (Figure 4). Therefore, the turnaround time to culture result reporting tended to be slightly longer for negative samples (median 48 h, IQR 46 h 43 min–51 h 34 min) than for positive specimens (median 47 h 34 min, IQR 40 h 16 min–49 h 16 min), thought the difference was not significant (*p* = 0.33).

### 3.4. Xpert Diagnostic Performances

The results of Xpert testing procedure compared to the biological consensus, stratified per investigated pathogen, are presented in Table 1. Corresponding tables for SoC tests are provided in Appendix A (Table A2). Corresponding performance metrics with 95% CIs are summarised in Table 2. Overall, Xpert had all performance indicators above 94% compared to consensus. For each investigated pathogen, the associated sensitivity was ≥94%, specificity was ≥99%, PPV was ≥88% and NPV was ≥99%, evidencing the very high capacity of Xpert to identify reliably both positive and negative clinical specimens, with a very limited fraction of false negatives. As mentioned in Section 2, no positive samples were included in the study for Giardia and Cryptosporidium, therefore preventing the evaluation of sensitivity and PPV for those pathogens.

**Figure 5:**
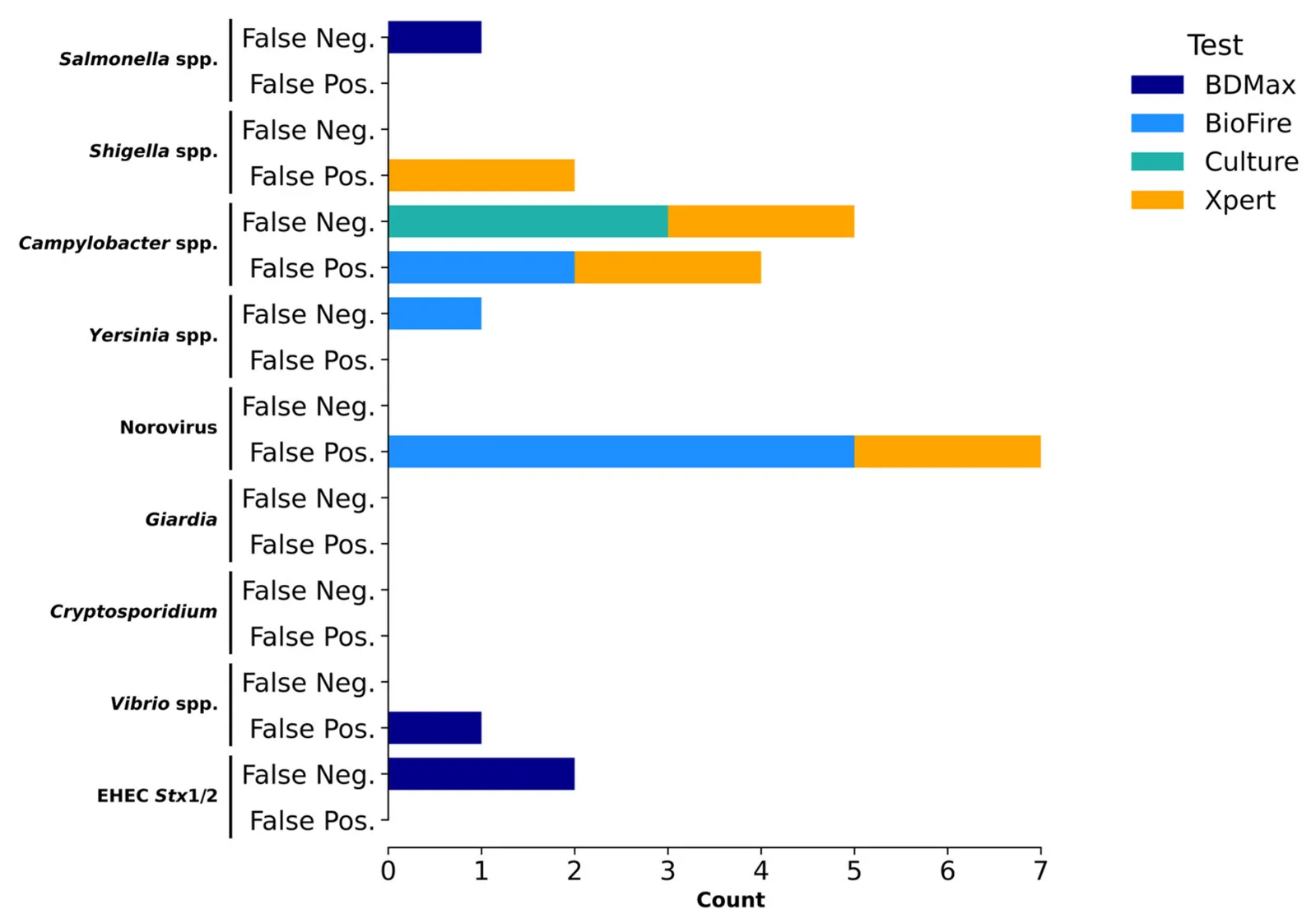
Discordant results by pathogen and SoC test platform.

Test results were considered discordant if they were not consistent with the biological consensus, resulting in 23 tests classified as either FP (14 specimens) or FN (nine specimens). For eight samples the Xpert result was different from the consensus, whereas eight discordant samples were identified for BioFire, four for BDMax and three for conventional stool culture (Figure 5, Table 3). Most discordant assays were seen for *Campylobacter* spp., with nine samples with wrong classification (5 FN, 4 FP), followed by Norovirus (0 FN, 7 FP), *Shigella* spp. (0 FN, 2 FP) and EHEC *Stx*1/2 (2 FN, 0 FP).

All in all, Xpert generated less misclassifications than SoC approaches (eight and 15 misclassifications, respectively), in particular for Norovirus and EHEC *Stx*1/2, while the number of false positives and false negatives were comparable in *Campylobacter* spp. However, Xpert raised slightly more false positives for *Shigella* spp. than SoC approaches.

**Figure A2:**
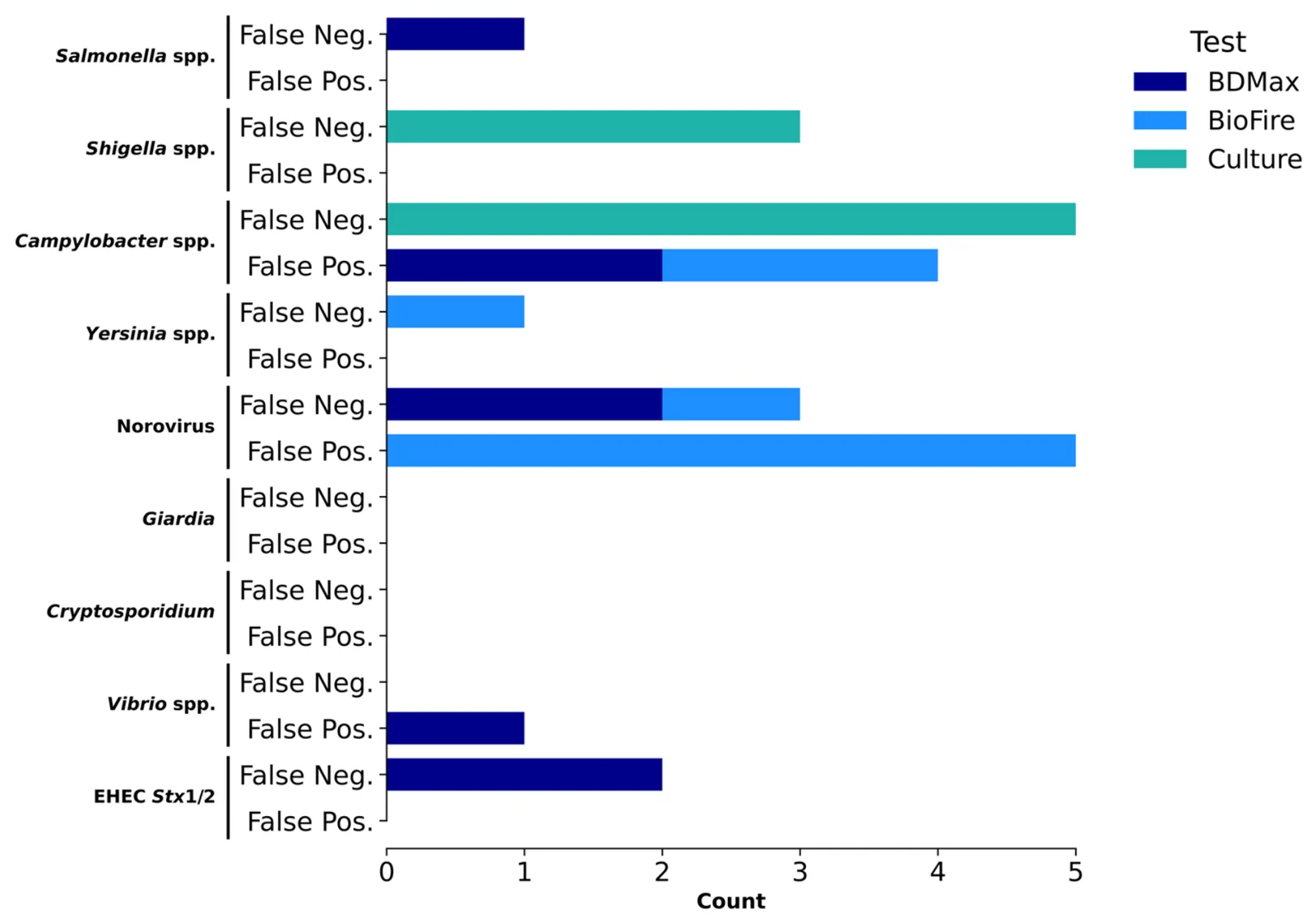
Discordant specimens between Xpert and SoC (irrespective of the biological consensus), per pathogen and SoC test type.

The contingency table between Xpert and SoC test results, as well as the associated comparative performance metrics (PPA, NPA and Kappa) with 95% CIs are presented in Appendix A (Table A3 and Table A4, respectively). Overall, PPA and NPA were ≥90%, and the Kappa value was 91%, showing the global agreement between Xpert and routine testing methods. At the level of each investigated pathogen, PPA was ≥86%, NPA was ≥99%, and Kappa was ≥86%. Details on concordant and discordant samples between Xpert and SoC testing procedures, overall and per SoC test type, are also shown in Appendix A (Table A1 and Figure A2).

## 4. Discussion

In this multicentre European study, the Cepheid Xpert GI RUO panel demonstrated excellent diagnostic performances for the detection of enteric pathogens directly from stool specimens. Compared with the consensus reference standard, overall sensitivity reached 98% and specificity exceeded 99%, while both PPV and NPV were above 94%. These performances are comparable to those of other syndromic PCR panels used for gastrointestinal pathogen testing [6,12].

The excellent performances observed across most investigated pathogens are consistent with previous evaluations of syndromic/multiplex gastrointestinal PCR panels, which have generally reported sensitivities and specificities above 90–95% for common bacterial and viral enteropathogens [3,13]. In our study, sensitivity reached 100% for *Salmonella* spp., *Shigella* spp., *Yersinia* spp., Norovirus, *Vibrio* spp. and EHEC *Stx*1/2, while only two false negative results were observed for *Campylobacter* spp. Importantly, the very high NPV observed for all pathogens may suggest that a negative Xpert result can reliably exclude infection with the investigated microorganisms. However, given that NPV evaluation is dependent on pathogen prevalence, our findings may be influenced by the limited number of positive observations for some of the investigated pathogens (e.g., *Vibrio* spp., *Yersinia* spp., *Cryptosporidium*, *Giardia*). If confirmed, this characteristic would be of particular clinical value in hospital settings where rapid patient management decisions are required, particularly in emergency and paediatric departments.

Besides its diagnostic accuracy, one of the main advantages of Xpert was the substantial reduction in analytical turnaround time. The median time from sample receipt at the workbench to result reporting was only 1 h and 18 min, instead of 3 h for the BDMax and 48 h or more for conventional stool culture due to the enrichment step. Moreover, whereas Xpert and BioFire are single random access tests, BDMax requires one to three strips for bacteria, virus and parasite panels in small batches of one to 12 strips per batch/run, with up to two runs performed simultaneously. Thus, some samples may need to wait for the next run if the two runs are already in process, and this workflow constraint could not be reflected by the analytical TAT measurements. The evaluation of real-world clinical benefits associated with reduced TAT was not part of the present study. Nevertheless, a future IVD version of the test, pending additional studies and regulatory clearance, may have important clinical implications, including earlier implementation of targeted antimicrobial therapy when appropriate, more timely initiation of infection prevention and control measures, ordering of additional paraclinical tests (e.g., abdominal ultrasound to detect appendicitis), reduced patient isolation time, and potentially shorter hospital stays [14,15,16]. However, these benefits may remain limited unless the pre-analytical and post-analytical phases are specifically addressed, as they are critical determinants of overall time to result and integral to diagnostic stewardship. Notably, the clinical impact of these potential advantages remains hypothetical and warrants confirmation in prospective studies assessing the complete diagnostic workflow, including pre-analytical and post-analytical turnaround times.

The study design also allowed for comparison of Xpert with heterogeneous standard-of-care (SoC) approaches currently used across European microbiology laboratories. While some participating centres had already implemented syndromic/multiplex molecular panels, others continued to rely primarily on conventional culture. Despite these differences, agreement between Xpert and routine testing methods remained high, with overall PPA and NPA above 90% and a Cohen’s kappa coefficient of 0.91, indicating almost perfect agreement. These findings suggest that Xpert performs consistently across different diagnostic environments and may represent an attractive alternative both for laboratories transitioning from culture-based workflows and for those already using syndromic/multiplex molecular testing. Our results are consistent with those reported for other commercially available syndromic gastrointestinal molecular assays, including the BioFire FilmArray GI Panel and the BDMax Enteric panels, which have consistently demonstrated PPA/sensitivity values generally above 95% and NPA/specificity above 97–99% for most targets in multicentre clinical evaluations [6,17].

However, the heterogeneity of testing procedures across participating centres should also be considered when interpreting the statistical estimates. While some centres had already implemented syndromic/multiplex molecular panels, others primarily relied on conventional culture, reflecting the diversity of routine diagnostic practices across European microbiology laboratories. No statistical adjustment of diagnostic performance estimates according to testing site or centre was performed. Consequently, the overall sensitivity, specificity, PPA and NPA estimates may partly reflect between-centre differences in patient populations, pathogen prevalence, testing procedures and reference strategies, in addition to the intrinsic performance of the Xpert assay. This may be particularly relevant for less frequently detected pathogens. Nevertheless, the high overall agreement observed across centres suggests that these differences did not substantially alter the main conclusions of the study. The multicentre design and inclusion of different clinical settings should therefore be considered both a strength, by reflecting real-world diagnostic diversity, and a limitation when interpreting pooled performance estimates.

Analysis of discordant results provided additional insight into assay performances. Most discordances involved *Campylobacter* spp., which also represented the most frequently detected bacterial pathogens in the cohort. *Campylobacter* detection is known to be challenging because of its fastidious growth requirements [18], and the ability of molecular assays to detect low bacterial loads, prolonged post-infectious shedding, asymptomatic carriage, or residual DNA from non-viable organisms [19,20,21]. Furthermore, because *Campylobacter upsaliensis* is included among the BioFire GI Panel targets but not detected by the other assays, some discordant *Campylobacter*-positive results may reflect differences in target coverage rather than reduced assay specificity (in particular the two *Campylobacter* detections with BioFire identified as FP, see Table 3). In addition, *Campylobacter* may enter a viable-but-non-culturable state [22], leading to positive molecular results despite negative culture findings. Therefore, some molecular-positive/culture-negative discordances may reflect the persistence of bacterial nucleic acids rather than active infection or true false positive results.

Differences in target composition between the BDMax, BioFire and Xpert panels definitely represent a methodological consideration when interpreting discordant results. These differences, which are described in the Section 2, could theoretically introduce classification bias into pathogen-specific analyses, particularly for *Campylobacter* spp. (as stated above) and *Vibrio* spp. For *Vibrio* spp., the potential impact appears limited, as only one discordant sample was observed: it was positive with BDMax and negative with Xpert, with the negative result confirmed by BioFire. For *Campylobacter* spp., the prevalence of *C. upsaliensis* is reported to be very low in France and Germany (<0.5%) [23], making such a situation unlikely in the present study setting. Overall, although differences in target coverage should be acknowledged as a potential source of misclassification, their impact on the present estimates appears limited.

Similarly, the few false positive *Shigella* results observed with Xpert may reflect the well-described genetic overlap between *Shigella* spp. and enteroinvasive *Escherichia coli* (EIEC) [24]. Differences in the molecular targets used across assays may also contribute to discordant results, as target specificity influences the discrimination of *Shigella* from closely related enteric organisms. Nevertheless, the absolute number of discordant results remained very low and did not materially affect overall performance estimates.

The microbiological epidemiology observed in the present study was broadly consistent with the expected distribution of enteric pathogens in European healthcare settings [25,26,27]. Positivity rates varied across pathogens, reflecting both true epidemiological differences and the centre-specific pathogen screening profile for the present study. The predominance of *Campylobacter* spp., Norovirus and *Salmonella* spp. observed in our study mirrors the epidemiology reported by European surveillance networks, where Campylobacteriosis and Salmonellosis represent both the most commonly reported enteric bacterial infections and Norovirus remains a leading cause of acute gastroenteritis outbreaks after Rotavirus. Co-detections were identified in approximately 10% of positive specimens, most commonly involving *Campylobacter* spp. and Norovirus. This observation illustrates one of the major strengths of syndromic molecular panels compared with conventional methods, namely their ability to identify multiple pathogens simultaneously. Similar frequencies of co-detections have been reported in previous evaluations of gastrointestinal PCR panels, with mixed bacterial–viral infections representing a substantial proportion of positive specimens [14,15]. However, the clinical significance of co-detections remains uncertain. Detection of pathogen nucleic acids does not necessarily establish causality, as positive molecular results may reflect asymptomatic carriage, prolonged shedding following a resolved infection, or incidental detection of organisms not responsible for the patient’s current symptoms [4,15]. Consequently, multiplex PCR results should always be interpreted in conjunction with the patient’s clinical signs and epidemiological context.

The demographic characteristics of the study population also represent an important consideration when assessing the generalizability of these findings. The multicentre design and inclusion of patients from different age groups and clinical settings, together with the relatively balanced distribution between sexes, represent strengths of the study and contribute to the representativeness of the cohort. However, the age distribution should be interpreted cautiously, as patients aged ≥ 50 years constituted the majority of the study population. In particular, the relatively small proportion of children aged 0–5 years reflects the case mix of the participating hospitals, as recruitment was not specifically targeted toward paediatric patients. Therefore, the study findings may not be fully generalisable to all age groups, particularly young children, in whom the epidemiology and distribution of enteric pathogens may differ. Conversely, the predominance of older adults may have influenced the observed pathogen prevalence and consequently the predictive values of the assays.

Several additional limitations should be acknowledged. First, the prevalence of some pathogens was low, resulting in wide confidence intervals around sensitivity estimates. No positive specimens for *Giardia* or *Cryptosporidium* were identified, preventing assessment of Xpert sensitivity for these targets. This finding likely reflects the epidemiological profile of the study population, which consisted predominantly of adult inpatients and outpatients in western European care centres, a setting in which these parasitic pathogens are less commonly encountered. In addition, the study was conducted during the winter season, a period generally associated with a higher prevalence of bacterial gastroenteritis. Consequently, viral gastroenteritis cases may have been underrepresented, as these infections tend to occur more frequently during the summer months. Moreover, the Xpert GI (RUO) panel could not detect Rotavirus and Adenovirus, which are very frequently involved in viral gastrointestinal infections. Similarly, only a small number of positive samples were available for *Vibrio* spp., *Yersinia* spp. and EHEC *Stx*1/2.

Second, the absence of a universally accepted gold standard applicable to all investigated pathogens and samples across participating centres required the use of a composite molecular consensus approach based on the “2-out-of-3” rule, similar to previous diagnostic evaluation studies [3,13]. In the present study, this rule was applied only to discordant samples and was therefore equivalent, in practice, to considering the result of the third test as the consensus or biological truth when the other two tests disagreed. Although this approach is supported by the use of validated and commercially approved assays as reference tests, several sources of bias cannot be excluded. Because molecular assays contributed to the definition of the reference result, incorporation bias is possible. In particular, several recent studies have reported unexpectedly high false positive rates for Norovirus detection with the BioFire panel [3,28], leading to manufacturer-issued safety notices and confirmatory testing recommendations.

In addition, only discordant samples were re-verified, while concordant samples were not independently re-tested. Consequently, a sample for which SoC and Xpert results were concordant but both differed from the true biological status would not have been identified, potentially resulting in misclassification and biased performance estimates. We considered this risk to be limited because the SoC methods used in the study were validated and commercialised assays widely used in routine hospital procedures and expected to have high diagnostic performance. Nevertheless, this limitation should be considered when interpreting the reported accuracy estimates.

The reference approach also differed between centres, as different assays could be used as the third validation test. Together with differences in target composition between the Xpert, BDMax and BioFire panels, this may have introduced differential classification of samples and affected pathogen-specific statistical estimates. The specific genetic markers used by the different commercial assays cannot be disclosed because they are subject to proprietary and intellectual property restrictions. The pathogen targets covered by each panel, which are relevant to the interpretation of discordant results, are described in Section 2. In addition, no adjustment for testing site or centre was performed in the statistical analyses. The pooled performance estimates may therefore partly reflect centre-related differences in patient characteristics, pathogen prevalence, testing procedures and reference strategies. Although these sources of heterogeneity are inherent to a multicentre evaluation conducted under diverse real-world conditions, they should be considered when interpreting the reported estimates. Nevertheless, the limited number of discordant observations for most pathogens and the high overall agreement across centres suggest that these methodological differences are unlikely to have substantially modified the main conclusions of the study.

In addition, this study was not designed to capture clinical outcome data such as changes in antimicrobial treatment, length of hospital stays, additional paraclinical tests or infection control decisions following test results. The clinical and health economic impact of Xpert implementation therefore remains to be assessed in dedicated prospective studies.

Overall, our findings indicate that the Xpert GI (RUO) panel provides rapid and highly accurate detection of major bacterial and viral enteropathogens. Its performance was comparable to, and in some instances slightly better than, existing routine diagnostic approaches while substantially reducing turnaround time. Despite the methodological limitations inherent to the use of heterogeneous SoC procedures and a composite molecular reference approach, the high level of agreement observed across centres supports the robustness of the overall findings. These characteristics could support the integration of a future IVD version of this test into routine diagnostic workflows for patients with suspected acute infectious gastroenteritis, notably in the emergency room, and warrant further studies evaluating its clinical and economic impact in real-world settings.

## Figures and Tables

**Table 1:** Contingency table between Xpert and consensus results, stratified per pathogen.

Pathogen	Consensus POS	Consensus NEG	Xpert TP	Xpert FN	Xpert FP	Xpert TN
*Salmonella* spp.	25	481	25	0	0	481
*Shigella* spp.	14	493	14	0	2	491
*Campylobacter* spp.	37	471	35	2	2	469
EHEC *Stx*1/2	15	300	15	0	0	300
*Vibrio* spp.	3	312	3	0	0	312
*Yersinia* spp.	8	380	8	0	0	380
Norovirus	26	159	26	0	2	157
*Giardia*	0	118	0	0	0	118
*Cryptosporidium*	0	118	0	0	0	118
**All pathogens**	128	2832	126	2	6	2826

**Table 2:** Diagnostic performance metrics of Xpert compared to consensus (sensitivity, specificity, PPV and NPV, in %), with 95% CIs, stratified per pathogen.

Pathogen	Sensitivity (95% CI)	Specificity (95% CI)	PPV (95% CI)	NPV (95% CI)
*Salmonella* spp.	100 (86.7–100)	100 (99.2–100)	100 (86.7–100)	100 (99.2–100)
*Shigella* spp.	100 (78.5–100)	99.6 (98.5–99.9)	87.5 (64.0–96.5)	100 (99.2–100)
*Campylobacter* spp.	94.6 (82.3–98.5)	99.6 (98.5–99.9)	94.6 (82.3–98.5)	99.6 (98.5–99.9)
EHEC *Stx*1/2	100 (79.6–100)	100 (98.7–100)	100 (79.6–100)	100 (98.7–100)
*Vibrio* spp.	100 (43.9–100)	100 (98.8–100)	100 (43.9–100)	100 (98.8–100)
*Yersinia* spp.	100 (67.6–100)	100 (99.0–100)	100 (67.6–100)	100 (99.0–100)
Norovirus	100 (87.1–100)	98.7 (95.5–99.7)	92.9 (77.4–98.0)	100 (97.6–100)
*Giardia*	-	100 (96.8–100)	-	100 (96.8–100)
*Cryptosporidium*	-	100 (96.8–100)	-	100 (96.8–100)
**All pathogens**	98.4 (95.8–100)	99.8 (99.6–99.9)	95.5 (92.0–98.6)	99.9 (99.8–100)

**Table 3:** Details on the discordant results by pathogen and SoC test platform. Of note, two additional samples (with grey background in the table) for which no consensus could be obtained due to an insufficient amount of biological material to perform additional testing were added to the table compared to Figure 5.

Pathogen	SoC Type	SoC Result	Xpert Result	Consensus	N
*Salmonella* spp.	BDMax	NEG	POS	POS	**1**
*Shigella* spp.	Culture	NEG	POS	NEG	2
*Campylobacter* spp.	BDMax	POS	NEG	POS	2
*Campylobacter* spp.	BioFire	POS	NEG	NEG	**2**
*Campylobacter* spp.	Culture	NEG	POS	POS	**3**
*Campylobacter* spp.	Culture	NEG	POS	NEG	2
*Yersinia* spp.	BioFIre	NEG	POS	POS	**1**
Norovirus	BDMax	NEG	POS	NEG	1
Norovirus	BioFire	POS	NEG	NEG	**5**
Norovirus	BioFire	NEG	POS	NEG	1
*Vibrio* spp.	BDMax	POS	NEG	NEG	**1**
EHEC *Stx*1/2	BDMax	NEG	POS	POS	**2**
*Shigella* spp.	Culture	NEG	POS	Unknown	1
Norovirus	BDMax	NEG	POS	Unknown	1

## Data Availability

The data underlying this study are not publicly available because they contain personal health information and their sharing could compromise participant confidentiality. De-identified data may be made available by the corresponding author upon reasonable request, subject to institutional approval and applicable ethical and legal requirements. Similarly, code used to perform statistical analyses may be made available upon request, all corresponding demands must be addressed to contact@dali.science.

## Appendix A

**Table A1:** Number of concordant and discordant samples between Xpert and SoC, per pathogen and per SoC test type.

Organism	Xpert POS (All SoC)	Xpert/BioFire Concord.	Xpert POS/BioFire NEG	Xpert NEG/BioFire POS	Xpert/BDMax Concord.	Xpert POS/BDMax NEG	Xpert NEG/BDMax POS	Xpert/Culture Concord.	Xpert POS/Culture NEG	Xpert NEG/Culture POS
*Salmonella* spp.	25	2	0	0	19	1	0	3	0	0
*Shigella* spp.	17	0	0	0	12	0	0	2	3	0
*Campylobacter* spp.	37	4	0	2	25	0	2	3	5	0
EHEC *Stx*1/2	15	2	0	0	11	2	0	0	0	0
*Vibrio* spp.	3	0	0	0	3	0	1	0	0	0
*Yersinia* spp.	8	0	1	0	7	0	0	0	0	0
Norovirus	29	9	1	5	17	2	0	0	0	0
*Giardia*	0	0	0	0	0	0	0	0	0	0
*Cryptosporidium*	0	0	0	0	0	0	0	0	0	0
**NEGATIVE**	2830	1036			1148			636		
**TOTAL**	2964	1053	2	7	1242	5	3	644	8	0
Samples Tested	508	118	2	7	197	5	3	193	8	0

**Table A2:** Diagnostic performance metrics of SoC (overall and per SoC type) compared to consensus (sensitivity, specificity, PPV and NPV, in %), with 95% CIs, stratified per pathogen.

**SoC** **—All Tests Combined.**				
**Pathogen**	**Sensitivity** **(95% CI)**	**Specificity** **(95% CI)**	**PPV** **(95% CI)**	**NPV** **(95% CI)**
*Salmonella* spp.	96.0 (80.5–99.3)	100 (99.2–100)	100 (86.2–100)	99.8 (98.8–100)
*Shigella* spp.	100 (78.5–100)	100 (99.2–100)	100 (78.5–100)	100 (99.2–100)
*Campylobacter* spp.	91.9 (78.7–97.2)	99.6 (98.5–99.9)	94.4 (81.9–98.5)	99.4 (98.1–99.8)
EHEC *Stx*1/2	86.7 (62.1–96.3)	100 (98.7–100)	100 (77.2–100)	99.3 (97.6–99.8)
*Vibrio* spp.	100 (43.9–100)	99.7 (98.2–99.9)	75.0 (30.1–95.4)	100 (98.8–100)
*Yersinia* spp.	87.5 (52.9–97.8)	100 (99.0–100)	100 (64.6–100)	99.7 (98.5–100)
Norovirus	100 (87.1–100)	96.9 (92.9–98.6)	83.9 (67.4–92.9)	100 (97.6–100)
*Giardia*	-	100 (96.8–100)	-	100 (96.8–100)
*Cryptosporidium*	-	100 (96.8–100)	-	100 (96.8–100)
**All pathogens**	94.5 (91.3–98.4)	99.7 (99.5–99.9)	93.8 (89.0–97.6)	99.8 (99.6–99.9)
**SoC—BDMax**				
**Pathogen**	**Sensitivity** **(95% CI)**	**Specificity** **(95% CI)**	**PPV** **(95% CI)**	**NPV** **(95% CI)**
*Salmonella* spp.	95.0 (76.4–99.1)	100 (97.9–100)	100 (83.2–100)	99.4 (96.9–99.9)
*Shigella* spp.	100 (75.8–100)	100 (98.0–100)	100 (75.8–100)	100 (98.0–100)
*Campylobacter* spp.	100 (87.5–100)	100 (97.8–100)	100 (87.5–100)	100 (97.8–100)
EHEC *Stx*1/2	84.6 (57.8–95.7)	100 (98.0–100)	100 (74.1–100)	98.9 (96.2–99.7)
*Vibrio* spp.	100 (43.9–100)	99.5 (97.1–99.9)	75.0 (30.1–95.4)	100 (98.0–100)
*Yersinia* spp.	100 (64.6–100)	100 (98.0–100)	100 (64.6–100)	100 (98.0–100)
Norovirus	100 (81.6–100)	100 (92.9–100)	100 (81.6–100)	100 (92.9–100)
**All pathogens**	97.0 (93.9–100)	99.9 (99.7–100)	99.0 (96.7–100)	99.7 (99.5–100)
**SoC—BioFire**				
**Pathogen**	**Sensitivity** **(95% CI)**	**Specificity** **(95% CI)**	**PPV** **(95% CI)**	**NPV** **(95% CI)**
*Salmonella* spp.	100 (34.2–100)	100 (96.8–100)	100 (34.2–100)	100 (96.8–100)
*Shigella* spp.	-	100 (96.8–100)	-	100 (96.8–100)
*Campylobacter* spp.	100 (51.0–100)	98.2 (93.8–99.5)	66.7 (30.0–90.3)	100 (96.7–100)
EHEC *Stx*1/2	100 (34.2–100)	100 (96.8–100)	100 (34.2–100)	100 (96.8–100)
*Vibrio* spp.	-	100 (96.8–100)	-	100 (96.8–100)
*Yersinia* spp.	0.0 (0.0–79.3)	100 (96.8–100)	-	99.2 (95.4–99.9)
Norovirus	100 (70.1–100)	95.4 (89.7–98.0)	64.3 (38.8–83.7)	100 (96.4–100)
*Giardia*	-	100 (96.8–100)	-	100 (96.8–100)
*Cryptosporidium*	-	100 (96.8–100)	-	100 (96.8–100)
**All pathogens**	94.4 (82.2–100)	99.3 (98.8–99.7)	70.8 (50.0–88.9)	99.9 (99.7–100)
**SoC—Culture**				
**Pathogen**	**Sensitivity** **(95% CI)**	**Specificity** **(95% CI)**	**PPV** **(95% CI)**	**NPV** **(95% CI)**
*Salmonella* spp.	100 (43.9–100)	100 (98.0–100)	100 (43.9–100)	100 (98.0–100)
*Shigella* spp.	100 (34.2–100)	100 (98.0–100)	100 (34.2–100)	100 (98.0–100)
*Campylobacter* spp.	50.0 (18.8–81.2)	100 (98.0–100)	100 (43.9–100)	98.4 (95.5–99.5)
*Yersinia* spp.	-	100 (95.0–100)	-	100 (95.0–100)
**All pathogens**	72.7 (41.7–100)	100 (100–100)	100 (100–100)	99.5 (98.9–100)

**Table A3:** Contingency table between Xpert and SoC results per pathogen.

Pathogen	SoC POS	SoC NEG	Xpert POS SoC POS	Xpert NEG SoC POS	Xpert POS SoC NEG	Xpert NEG SoC NEG
*Salmonella* spp.	24	482	24	0	1	481
*Shigella* spp.	14	493	14	0	2	491
*Campylobacter* spp.	36	472	32	4	5	467
EHEC *Stx*1/2	13	302	13	0	2	300
*Vibrio* spp.	4	311	3	1	0	311
*Yersinia* spp.	7	381	7	0	1	380
Norovirus	31	154	26	5	2	152
*Giardia*	0	118	0	0	0	118
*Cryptosporidium*	0	118	0	0	0	118
**All pathogens**	129	2831	119	10	13	2818

**Table A4:** Table of PPA, NPA and Kappa when comparing Xpert to SoC (overall and per SoC type). Of note, no 95% CI could be assessed for Kappa in some cases, due to the limited number of positive samples (indicated by ** instead of 95% CIs). Estimates for all pathogens together are highlighted in bold.

**SoC** **—All tests combined**			
**Pathogen**	**PPA in % (95% CI)**	**NPA in % (95% CI)**	**Kappa (95% CI)**
*Salmonella* spp.	96.00 (80.46–99.29)	100 (99.21–100)	0.98 (0.93–1.00)
*Shigella* spp.	87.50 (63.98–96.50)	100 (99.22–100)	0.93 (0.82–1.00)
*Campylobacter* spp.	86.49 (72.02–94.09)	99.15 (97.84–99.67)	0.87 (0.78–0.95)
EHEC *Stx*1/2	86.67 (62.12–96.26)	100 (98.74–100)	0.93 (0.78–1.00)
*Vibrio* spp.	100 (43.85–100)	99.68 (98.21–99.94)	0.86 (**)
*Yersinia* spp.	87.50 (52.91–97.76)	100 (99.00–100)	0.93 (0.74–1.00)
Norovirus	92.86 (77.35–98.02)	96.82 (92.76–98.63)	0.86 (0.74–0.96)
*Giardia*	-	100 (96.85–100)	-
*Cryptosporidium*	-	100 (96.85–100)	-
**All pathogens**	90.15 (85.40–95.22)	99.65 (99.41–99.83)	0.91 (0.87–0.94)
**SoC—BDMax**			
**Pathogen**	**PPA in % (95% CI)**	**NPA in % (95% CI)**	**Kappa (95% CI)**
*Salmonella* spp.	95.00 (76.39–99.11)	100 (97.88–100)	0.97 (0.91–1.00)
*Shigella* spp.	100 (75.75–100)	100 (97.97–100)	1.00 (1.00–1.00)
*Campylobacter* spp.	100 (86.68–100)	98.84 (95.86–99.68)	0.96 (0.88–1.00)
EHEC *Stx*1/2	84.62 (57.77–95.67)	100 (97.95–100)	0.91 (0.77–1.00)
*Vibrio* spp.	100 (43.85–100)	99.48 (97.14–99.91)	0.85 (**)
*Yersinia* spp.	100 (64.57–100)	100 (98.02–100)	1.00 (1.00–1.00)
Norovirus	94.44 (74.24–99.01)	100 (92.73–100)	0.96 (0.87–1.00)
**All pathogens**	95.92 (92.13–99.05)	99.74 (99.40–100)	0.96 (0.93–0.99)
SoC—BioFire			
**Pathogen**	**PPA in % (95% CI)**	**NPA in % (95% CI)**	**Kappa (95% CI)**
*Salmonella* spp.	100 (34.24–100)	100 (96.79–100)	1.00 (**)
*Shigella* spp.	-	100 (96.85–100)	-
*Campylobacter* spp.	100 (51.01–100)	98.25 (93.83–99.52)	0.79 (**)
EHEC *Stx*1/2	100 (34.24–100)	100 (96.79–100)	1.00 (**)
*Vibrio* spp.	-	100 (96.85–100)	-
*Yersinia* spp.	0.00 (0.00–79.35)	100 (96.82–100)	0.00 (**)
Norovirus	90.00 (59.58–98.21)	95.37 (89.62–98.01)	0.72 (0.47–0.91)
*Giardia*	-	100 (96.85–100)	-
*Cryptosporidium*	-	100 (96.85–100)	-
**All pathogens**	89.47 (75.57–100)	99.33 (98.76–99.71)	0.79 (0.63–0.92)
SoC—Culture			
**Pathogen**	**PPA in % (95% CI)**	**NPA in % (95% CI)**	**Kappa (95% CI)**
*Salmonella* spp.	100 (43.85–100)	100 (98.00–100)	1.00 (**)
*Shigella* spp.	50.00 (15.00–85.00)	100 (98.00–100)	0.66 (**)
*Campylobacter* spp.	37.50 (13.68–69.43)	100 (97.97–100)	0.53 (0.00–0.85)
*Yersinia* spp.	-	100 (95.00–100)	-
**All pathogens**	53.33 (22.27–80.0)	100 (100–100)	0.69 (0.40–0.87)
